# New Remedies

**Published:** 1887-08

**Authors:** 


NEW REMEDIES.

There has been a disposition on the part of some to multiply remedies, and to abandon old and well-known preparations for others which were comparatively untried, and which had nothing but novelty to recommend them. Cautious experimentation with new drugs is one thing, and the unqualified commendation of articles new to the pharmacopœia is quite another. Dentists should become acquainted with new remedies, for a valuable adjunct in practice may thus be secured. But to recommend to those who obtain their ideas at second hand that they should abandon the use of a preparation having recognized virtues for another of only supposed efficacy, is scarcely excusable.

Iodol, for instance, has been warmly advocated as a substitute for iodoform. It was asserted by medical men that it possessed all the good qualities of the latter without its disadvantages; that Iodol was but the concentrated essence of iodoform, divested of its evil odor and irritative characteristics. Further experimentation has taught that it is not at all well adapted for the offices of iodoform, and it has as rapidly fallen out of favor as it grew into it at first.

Eugenol, again, is not at all the same thing with oil of cloves, nor will it take the place of the latter. That it possesses qualities which sometimes make it useful in dental and medical practice is not for a moment denied, but it has none of the soothing and obtunding characteristics of the oil of cloves, and widely differs from it physically.

Oil of Eucalyptus is another disappointment. Those who have attempted to employ it in place of carbolic acid or iodoform as an antiseptic, have been disappointed. It possesses a high odor and masks the fetor of septicism, but it is not, at least in our hands, a satisfactory disinfectant, or even antiseptic.

Sanitas Oil is, perhaps, even a worse disappointment than either of the others. It is a proprietary, we believe even a "quack" preparation, that originated in England, and it has been widely and persistently advertised as a disinfectant for cess-pools, and for sanitary uses. For these purposes it may be sufficient, though we should think that its vile odor would be enough to condemn it, but for dental purposes it does not to us seem at all adapted.

Our materia medica is all too limited, yet it is unwise to enlarge it by introducing drugs of not only doubtful utility, but those which are so far untried that they are liable to prove positively deleterious.

